# The impact of different boron levels in diet on performance and eggshell quality of Japanese quails (*Coturnix japonica*)

**DOI:** 10.1016/j.sjbs.2021.10.046

**Published:** 2021-10-25

**Authors:** Muhammet Ali Kara

**Affiliations:** Siirt University, Department of Animal Science, Siirt, Turkey

**Keywords:** Boron, Eggshell quality, Performance, Ration

## Abstract

This study was carried out to investigate the effects of different levels of boron supplementation on the performance and eggshell quality of 162 breeding quails at 7 weeks of age. For this purpose, 9 different diets with addition of 0, 20, 30, 40, 60, 80, 120, 160 and 240 mg/kg B were used. The experiment was designed at 9 different levels of B, as 3 replicates, on 27 subgroups in total. Each cage compartment was accepted as replicate and 4 female and 2 male quails were placed in each cage. Water and feed are provided as ad-libutum to the quails that are housed in battery-type cages and the lighting was applied continuously. The final body weight (FBW) of the quails, body weight gain (BWG), feed intake (FI), egg production (EP%), egg mass (EM), feed conversion ratio (FCR), egg weight (EW), egg specific gravity (ESG), egg surface area (ESA), eggshell weight (ESW%), eggshell breaking strength (ESBS) values were measured. The effect of the addition of different levels of B to the rations on BW, BWG, FI, EP %, EM and FCR was not statically significant, while effects on EW (p < 0.01), ESG (p < 0.01), ESA (p < 0.05), ESR % (p < 0.01) and ESBS (p < 0.01) was found to be significant. As a result, the 20 mg/kg B addition to the rations was sufficient for a good eggshell quality and performance, and it was observed that higher doses had a negative effect on performance and shell quality in general.

## 1 Introduction

Studies on the improvement of factors affecting eggshell quality in poultry breeding are of great importance for the commercial egg industry because defects in shell quality can cause significant economic losses. In many studies, it has been reported that approximately 10 to 15% of all losses in egg production is directly related to eggshell quality (Stefanello et al., 2014). Additionally, high eggshell fracture strength, and a perfect shell formation prevent the penetration of pathogenic bacteria such as *Salmonella* sp. into the egg (Świątkiewicz et al., 2010). Studies on the effect of nutrition on eggshell quality in laying hens are mostly focused on macro minerals such as Ca and P and vitamin D3 (Nys, 1999, Neijat et al., 2011). Nonetheless, it is now known that trace minerals have an important role in the mineralization process. Trace minerals are essential in the rations of the laying hens as they participate in the biochemical events necessary for normal growth and development during the formation of bones and eggshells and in the development of embryos (Richards et al., 2010).

Boron (B) is not found as free-state in nature and it is a trace element found as borates, in the form of compounds formed as a result of the combination with oxygen. Boron is an essential element for plants and, in agricultural production, the presence or concentration of B in soil and irrigation water is an important factor affecting the quantity and quality of crops (Tanaka and Fujiwara, 2008). While satisfactory results have not been obtained by the initial studies to show that boron can be essential for human and animals (Nielsen, 2008), a considerable amount of information has been obtained in studies conducted since the 1980s, and the element is shown to be possibly essential for frogs (Fort et al., 1998), fish (Rowe et al., 1998), and human (Nielsen, 1994, Hunt, 1994, Hunt et al., 1997). Boron is an essential element reported by the World Health Organization (WHO, 1998) for human, and studies on human and animals have shown that it may be important for mineral metabolism, brain function, hormone regulation, and prevention of osteoporosis. (Khaliq et al., 2018, Abdelnour et al., 2018). Boron has been reported to affect bone development and mineralization in association with its interactions with Ca, P and Mg minerals. (Chapin et al., 1997; Armstrong, 2000). In studies conducted with laying hens, it was stated that the addition of boron to the ration increased the bone-breaking force, prevented joint disorders and improved the internal and external quality of the eggs (Mızrak et al., 2008, Koçbeker et al., 2017).

The aim of this study was to investigate the effect of different levels of B supplement on the performance and eggshell quality in breeding quails.

## 2 Materials & methods

### 2.1 Animals

In this study, 162 (7 weeks old) breeding quails with a male-to-female ratio of 1/2 were used. The quails were selected after brood of a sufficient number of eggs were collected from the breeding flock. In this framework, newly born quails were grown for 6 weeks and selected based on similar live weight range.

### 2.2 Feed

Diets of experiment were prepared by taking existing feed materials and the required raw materials (such as manganese trace mineral mixture, demineralized vitamin mix) obtained from the local market into consideration. The nutrient contents of the raw materials used in the ration formulation are given in Table 1.

**Table 1:** Nutrient composite on of raw materials used in main ration.

**Raw material**	**HP** **(%)**	**ME (kcal/kg)**	**Ca** **(%)**	**Total P** **(%)**	**Lysine** **(%)**	**Methionine** **(%)**	**Cystine** **(%)**
Barley	11.00	2761	0.07	0.40	0.20	0.20	0.24
Corn	8.80	3300	0.03	0.27	0.20	0.18	0.15
Soybean meal	47.03*	2254	0.28	0.66	2.70	0.69	0.67
Sunflower seed meal	36.02*	2018	0.4	1	1.28	0.60	0.56
Vegetable oil	–	9000	–	–	–	–	–
Marble powder	–	–	37.5	–	–	–	–
DCP	–	–	24	18	–	–	–

* This was found as a result of the analysis.

### 2.3 Method

The study was conducted at Research and Application farm of Faculty of Agriculture, Department of Animal Science, Selçuk University, Turkey. The experiment was carried out at 7 weeks old 162 breeding quails with a male-to-female ratio of ½ for 7–28 weeks’ period. In the experiment, 9 different types of ration including 0, 20, 30, 40, 60, 80, 120, 160 and 240 mg/kg B were used. B was added to the ration as boric acid. All experimental rations were established in accordance with the recommendations (NRC, 1994) for breeding quails (Table 2). The experiment was carried out in 9 different B levels with 3 replications. 4 female + 2 male quails were placed in each cage and every cage was considered as replication.

**Table 2:** Ingredients and calculated nutrient composition of the basal standard diet used in the experiment.

**Ingredients**	**%**
Barley	7.20
Corn	44.3
Soybean meal	28.6
Sunflower seed meal	6.0
Fat	5.3
Calcium oxide	6.2
DCP	1.4
Salt	0.40
L-Lysine	0.10
DL-methionine	0.15
Vitamine Premix^1^	0.25
Mineral Premix^2^	0.10
**Total**	**100**
**Nutrient composition**	
Energy (kcal ME/kg)	2903.3
HP (%)	20.03
Ca (%)	2.78
Ca (%*)	2.74
Total P (%*)	0.71
Usable P (%)	0.39
Lysine (%)	1.05
Methionine (%)	0.48
Methionine + Cystine (%)	0.79
Boron (mg/kg*)	14.18
Zinc (mg/kg*)	33.58

* Values were obtained from results of the analysis in ICP.

1 Vitamin premix in 1 kg of the ration provides: vitamin A, 8800 IU; vitamin D_3_, 2200  IU; vitamin E, 11 mg; nicounic acid, 44 mg; Cal-D-Pant., 8.8 mg; riboflavin 4.4 mg; thiamine, 25 mg; vitamin B_12_, 6.6 mg; folic acid, 1 mg; D-Biotin, 0.11 mg; choline, 220 mg

2 Mineral premix (zinc free) 1 kg of the ration provides: mangan, 80 mg; iron, 60 mg; copper, 5 mg; cobalt, 0.2 mg; iodine, 1 mg; selenium, 0.15 mg.

### 2.4 Determination of performance characteristics

The live weights of the birds were recorded as group weighing at the beginning and end of the experiment. The feed consumption of the animals was determined for 28 day periods for each group and daily average feed consumption was calculated. Eggs were collected daily and recorded, and thus egg yields were calculated. For the egg weight, all eggs were collected in the last 3 days of each 28 days’ period and were weighed on a 0.1 g precision scale. For the egg mass, the average egg yield per unit (amount/day) was multiplied by the egg weight (egg mass, g = (total number of eggs/28 days/number of animals) × average egg weight, g).

Feed conversion ratio was calculated by dividing the daily average feed consumption per quail to the egg mass (feed consumption, g/egg mass, g).

### 2.5 Determination of eggshell characteristics

Ten eggs were randomly collected in the last three days of 28 days in each of the groups and evaluated for egg specific gravity, shell fracture strength, and shell weight. To determine the egg specific gravity, the selected eggs weights in the air and in the water were determined by a suitable mechanism placed on the precision scale. After this measurement, specific weights are calculated according to the principle of Archimedes, with the following formula (Voisey and Hunt, 1973).

Egg Specific gravity (g / cm^3^) = (egg weight in air, g) / (the difference between the weight of the egg in air and water, g).

The fracture strength of eggs, whose specific gravities were determined, were measured in the fracture tester (Egg Force Reader, Orka Food Technology Ltd., Ramat Hasharon, Israel). After washing the shells of these eggs with tap water, they were dried at room temperature for three days and the shell weight was determined by weighing at 0.1 g precision scale.

The shell rate was calculated as% = (shell weight, g / egg weight, g) × 100.

### 2.6 Statistical analysis

In order to determine whether the effects of the applications on the examined parameters were significant, all data were analyzed by using the statistical package program STATISTICA 13.6. (Statistica) In cases F values were significant, Duncan's multiple comparison test was used to detect the differences between means (Duncan, 1955). It is necessary to check whether the homogeneity of variances and normality assumptions are met before performing variance analysis. For this purpose, Kolmogorov-Smirnov (K-S) test was used for normality and Levene’s test was used for testing the homogeneity of variances. The p-values of the Levene and K-S tests showed non-significant results; thus, both assumptions were met. The total variance was partitioned into factors according to the following model:

$$Y_{\mathrm{ij}}=\;\mu \;+\;B_{i}+\;e_{\mathrm{ij}}$$

Where:

Y_ij_ = observation j in treatment I, µ = overall mean, B_i_ = effect of treatments (boron) add here the restrictions terms also and E_ij_ = experimental error add here the restrictions terms also.

## 3 Results

### 3.1 Performance characteristics

The effect of different levels B addition on performance are given in Table 3. At the beginning of the experiment body weights of the quails did not differ between treatments. This results showed that the quails were homogeneously distributed in the experimental groups. In the present study BW, BWG, FI, FCR, EM and EP% for breeding quails during the whole experiment was not affected by boron supplementation (Table 3). Although the BWG was not statistically significant from the boron levels, a numerical increase was observed in the BWG in the groups added 160 and 240 mg/kg B. However, the differences between the groups for egg weight was statistically significant (p ≤ 0.01). Accordingly, the highest egg weight (13.421 g) was obtained from the control group and it was the highest compared with other groups, except for the group containing 20 mg/kg B. Thus, as the level of B increases, it is clear that the weight of the eggs decreases even if this variation is not linear.

**Table 3:** The Effect of Different Levels of Boron Addition on Performance.

**B levels** **mg/kg**	**IBW** **(g)**	**FBW** **(g)**	**BWG** **(g)**	**FI** **(g/bird/day)**	**FCR**	**EM** **(g/bird/day)**	**EP** **(%)**	**EW** **(g)**
0	196.72	222.18	25.46	29.100	2.757	10.859	80.774	13.421a
20	199.22	232.17	32.94	29.517	2.902	10.497	79.940	13.184ab
30	196.55	225.28	28.72	29.242	2.793	10.593	82.381	12.897 cd
40	196.17	235.94	39.78	28.277	2.611	11.131	86.488	12.929bcd
60	205.83	232.28	26.45	29.574	2.785	10.685	84.107	13.053bc
80	191.11	227.46	36.34	27.725	4.025	8.854	86.070	12.832cde
120	199.94	234.39	34.44	29.012	2.857	10.766	81.964	12.550ef
160	195.33	236.72	41.39	29.745	2.840	10.773	84.881	12.667def
240	189.72	233.78	44.05	29.272	2.920	10.479	82.976	12.447f
SEM	3.65	3.63	4.73	0.828	0.490	0.967	4.647	0.101
P-value	0.1634	0.1637	0.1538	0.7481	0.6712	0.8701	0.9783	0.0001

IBW: Initial body weight; FBW: Final body weight; BWG: Body weight gain; FI: Feed intake; FCR: Feed conversion ratio; EM: Egg mass; EP: Egg Production; EW: Egg weight

### 3.2 Eggshell quality criteria

Eggshell quality parameters are presented in Table 4. The effects of levels of boron on the ESG (p ≤ 0.01), ESA (p ≤ 0.05), ESW (p ≤ 0.01) and ESBS (p ≤ 0.05) were statistically significant. The highest ESG value of 1.0742 was obtained from the control group. The ESG of this group was found to be higher than that of all other groups, except for the groups fed with basal diet containing 20 to 60 mg/kg B. The ESA value was observed to be highest in the quails fed with control diet, and this value was significantly higher than that of the diets with 80, 120, 160 and 240 mg/kg B. In the current study, the ESW % value of the quails fed by ration containing 20 mg/kg B was different in compared to quails fed by the rations containing other levels of B. The ESW % of quails fed with rations containing 20 mg/kg B was significantly higher than that of all other quails, except for the quails fed with rations containing 0 and 80 mg/kg B. At the same time, ESBS was the highest in the quails fed with rations containing 20 mg/kg B, except for the quails fed with rations containing 0 mg/kg B.

**Table 4:** The Effect of Different Levels of Boron Addition on Egg Shell Quality.

**B, mg/kg**	**ESG**	**ESA (cm^2^)**	**ESR (%)**	**ESBS (N)**
0	1.0742a	24.536a	8.660ab	1.637ab
20	1.0726ab	24.474a	8.802a	1.745a
30	1.0709bc	24.268ab	8.141ef	1.529bcd
40	1.0705c	24.152ab	8.318de	1.467d
60	1.0725ab	24.090ab	8.408 cd	1.504 cd
80	1.0702c	23.497bc	8.663ab	1.455 cd
120	1.0702c	23.435bc	8.070f	1.461d
160	1.0630d	23.411bc	8.187def	1.485dc
240	1.0714bc	22.779c	8.561bc	1.604bc
SEM	0.0007	0.2838	0.0733	0.0419
P-value	0.0001	0.0138	0001	0.0041

ESG: Egg specific gravity; ESA: Egg surface area; ESR: Eggshell ratio; ESBS: Eggshell breaking strength.

## 4 Discussion

### 4.1 Performance parameters

Evaluated studies on laying hens reported that B supplementation up to 250 ppm does not have a negative impact on BW. Pradhan et al. (2020) reported that there was an increase in performance indices of the broiler chicken with increased level of boron supplementation from 25 to 100 mg/kg diet. Similarly, El-Saadany et al. (2017) indicated that supplementation of boron in laying hens improved egg quality, reproductive and physiological status. Özdemir et al. (2016) reported that adding boron to diet in quail led partly to improvements. Koçbeker et al. (2017) reported that supplementation Ca and B in laying hens in pre-laying period did not affect the performance characteristics significantly. Again, Olgun et al. (2009) reported that the addition of B up to 0–300 mg/kg in laying hens did not significantly affect BW at the initial and at the end of the experiment, whereas in an another study the supplement of B level 0, 75 and 150 mg/kg caused a decrease in BW (Küçükyılmaz et al., 2014). In contrast to these findings, it was clearly indicated that BW in quails was not affected by the supplement of B showing that it was in accordance with our findings (Table 2).

Yeşilbağ and Eren (2008) reported that the addition of increased levels (0, 25, 50, 100 mg/kg) of B to the diets of aged hens caused a significant increase in FI. Nonetheless, Hakan et al. (2012) observed that the addition of different levels of B in laying hens for 1–14 week periods did not cause a significant difference in FI, although there was a significant decrease in FI at 5–6 weeks period. In another study (Küçükyılmaz et al., 2014), an important decrease was observed in FI with increasing B levels (0, 75 and 150 mg/kg). Olgun et al. (2013) showed that feeding with a level of 240 mg/kg B in laying hens was led to a statistically significant decrease in FI. However, Mızrak et al. (2010) did not detect any significant difference for FI.

Many studies have shown that adding different levels of B to the diet did not affect FCR, in line with our findings (Kurtoğlu et al., 2002, Yeşilbağ and Eren, 2008, Olgun et al., 2009, Mızrak et al., 2010). In contrast, some studies indicated that FCR was affected from the added B in some genotypes such as Japanese quails (Ayasan et al., 2011) and hens (Olgun et al., 2013, Küçükyılmaz et al., 2014) compared with control group. Mızrak et al., 2010, Ayasan et al., 2011 were indicated that adding of different levels of B to the diet of quails the EM did not affect in line with our finding. However, Olgun et al., 2009, Olgun et al., 2013 showed that the addition of B to the ration significantly decreased EM in laying hens. In our study, there were non-significant differences among groups in breeding quails fed with different levels of B for EP. Similar findings were shown by other studies (Wilson and Ruszler, 1998, Kurtoğlu et al., 2002, Yeşilbağ and Eren, 2008, Olgun et al., 2009, Mızrak et al., 2010, Ayasan et al., 2011).

In the present study, there was a significant decrease in EW with the increasing levels of B. These findings for EW were in accordance with results of the other studies (Olgun et al., 2009, Olgun et al., 2013, Küçükyılmaz et al., 2014, Ayasan et al., 2011). However, Yeşilbağ and Eren (2008) reported that there was an increase in EW as the level of B was raised. These findings for EW were in accordance with results of the other studies (Olgun et al., 2009, Olgun et al., 2013, Küçükyılmaz et al., 2014, Ayasan et al., 2011). Kurtoğlu et al., 2002, Mızrak et al., 2010 found no differences for EW in their studies. However, Yeşilbağ and Eren (2008) reported that there was an increase in EW at increasing level of B. The differences among findings mentioned above for performance parameters mainly can be resulted from species, genotype, age, the presence of antinutritional factors in addition to the B levels in the basal rations, B sources and levels used in rations, and the diverse nutrient compositions of the rations in comparison with other studies (Arslan and Macit, 2018).

### 4.2 Eggshell quality parameters

In our current study, ESG showed a decrease with increasing B levels. Similar results were reported by Olgun and Bahtiyarca (2015) on laying hens. However, in many studies it was indicated that the effect of different levels of B on the ESG was not statistically significant (Kurtoğlu et al., 2002, Eren et al., 2004, Olgun et al., 2009, Olgun et al., 2012). In the current study we found that increasing B levels decreased ESW%. Similarly, Küçükyılmaz et al. (2014) reported that the effect of adding different levels of B (0, 75 and 150 mg/kg) in laying hens was decreased ESW %. Nonetheless, Olgun et al., 2009, Olgun et al., 2012, Olgun and Bahtiyarca, 2015 detected no differences for this trait in laying hens. Again, we detected that effect of different levels of B addition on ESBS was statistically significant (Table 3). However, Arslan Kaya and Macit (2018) reported that the effect of different levels of B (0, 50, 75 and 150 mg/kg) on the ESBS in laying hens was not significant, although the addition of 50 mg/kg B in all groups caused an increase in ESBS. Yeşilbağ and Eren (2008) reported that the supplement of 25, 50 and 200 mg/kg B to the diet produced eggs with higher ESBS in healthy shell thickness and decreased eggshell deformation in laying hens. At the same time, Olgun and Bahtiyarca, 2015, Olgun et al., 2012, Olgun et al., 2009, Eren et al., 2004 reported that the addition of different levels of B in laying hens was not affect ESBS.

In poultry, it is desirable to have good shell quality in terms of production economy, and this should be followed closely during the production cycle. Genotype and age are the most influential factors in shell properties (Kemps et al., 2006). The possible causes of the inconsistency between the results of studies mentioned above may be the genotypes of the test materials, the age, the mineral composition in the nature of the rations and the differences in the tolerances of the animals against B when compared with results in the present study.

## 5 Conclusions

The study revealed that the addition of B to the diet did not significantly affect performance criteria such as CA, CAA, FI, FCR, EM and EP, except EW, but significantly affected eggshell quality criteria such as ESG, ESA, ESR, ESBS. Finally, it can be proposed that addition of 20 mg/kg B to the basal diet is beneficial for shell quality and performance in breeding quails, though the higher levels of B additions has generally a negative effect.

## Declaration of Competing Interest

The authors declare that they have no known competing financial interests or personal relationships that could have appeared to influence the work reported in this paper.
